# Cook County Hospital—Surgical Department

**Published:** 1876-07

**Authors:** 


### Hospitals.

### COOK COUNTY HOSPITAL—SURGICAL DEPARTMENT.

SERVICE OF DR. BOGUE.

(Reported by Dr. FENN.)

March 11, 1876. Dr. Bogue presented and made remarks on the following cases:

1. Dislocation of the right shoulder downward; four weeks standing; plump German; aged 45; anæsthetized slowly. The operation of reduction was begun in the usual way by placing the heel in the axilla and making traction; manipulation to cause free movement in the joint. It was not till after extreme and long continued exertion when it seemed that every attempt would fail, that the head of the bone was found to be in place. The arm was put in a sling and a wet compress ordered for the shoulder.

2. Operation for piles. A healthy, well developed young man; ether given by means of an enameled leather cup open at both ends and made to fit over the nose and mouth, filled with spongiopiline. Ether is thrown on sparingly through an opening in the cap of the bottle; anæsthesia was produced very slowly.

Dr. B. began the operation by inserting the two index fingers within the rectum. By pulling in opposite directions he dilated the sphincter sufficiently to obtain a fair view of the folds of hypertrophied membrane. Taking hold of these folds with ring forceps he brought them down one at a time, fixed on the clamps and destroyed them with the hot iron; sweet oil was smeared on the eschar; a wet compress was ordered, and opium if necessary.

March 14. 1. Crushed hand—loss of fingers. A ragged, dirty boy just brought in from a lumber mill in the vicinity. The remaining fragments of fingers were trimmed up with bone forceps. One slightly bleeding artery was ligated; oakum and bandage.

2. Necrosis of lower jaw. A little girl six years old. It has continued a year and a half; there is little doubt, therefore, that the necrosed bone is ready for removal. Ether was given. On examination and cutting, it was found that a piece of dead bone lay along the side of the jaw, probably separated for a year but held by thickened tissue; new bone has formed in abundance; dressed with a bit of sponge only to keep clean.

3. Anchylosis of the knee of long standing. Young man; has always gone on a crutch. The attempt is to be made to get the knee into a condition to be of use to him. If there is any motion it is very slight. The deformity from malposition of the head of the tibia and from the position of the leg at right angles to the thigh is considerable.

The preliminary operation will be the boring of the knee both through the head of the tibia and partially through the track of the joint, to induce softening; the object is ultimately to liberate the patella, and, if possible, straighten. After the boring, some tendons were partially divided. All efforts to straighten or to cause motion except in the slightest degree, failed. The knee was packed in oakum and wound with bandage to await results.

March 18. 1. Hydrocele injection. A fair looking young man. Disease began twelve months ago. The scrotum was punctured, fluid drawn off and the sac injected with compound solution of iodine in proportion of fifteen grains of iodine and thirty grains of iodide of potassium to one ounce of water. The application was evidently very painful, especially when the kneading began; fomentations ordered to be applied in a few hours when pain shall have subsided.

2. Ununited fracture of the tibia; injury last December. It has been bored once with no other result than to produce suppuration for a few days; boring a second time. No anæsthetic.

3. Street car injury. Boy 9 years old, just brought in; lacerated flesh wound of left leg, destroying the greater portion of the muscles of the calf, not opening joints; ligamentum patellæ exposed. The right tarso-phalangeal joint was laid open by a broad wound obliquely across the dorsum of the foot. An attempt was made to approximate the edges of the wound of the leg by a stitch or two in the lower portion. A weak solution of alcohol in water was applied over the wounds and the patient was ordered one grain of quinine three times a day, and milk; the leg to be placed in a sling.

4. Chronic caries of tibia, upper third. A young man apparently healthy. He says he received a charge of shot in his leg twenty years ago; he has been from place to place, and the attempt now to gouge out the decayed bone is only one of a series which have already been made. He will probably never get well.

March 21. 1. Anchylosis of the knee. Bored last week; no signs of inflammation. While ether was being given preparatory to some further trial, Dr. B. remarked concerning the improved method of giving ether, that it was both more economical and safer. The other day when this patient was first put to sleep, but three ounces of ether were used. By the usual way, a pound bottle full would have been consumed; of course the smaller the quantity the safer.

When anæsthesia was complete, the leg was brought over the end of the table, an assistant held the thigh firmly, and Dr. B. attempted to break up the adhesions, using an amount of force that seemed to threaten the popliteal vessels. By repeated attempts at both flexion and extension, the leg was finally carried through an arc of more than 45 degrees. A loud cracking sound greeted the ears of all who were in the room; blood flowed freely from the wounds of the former boring; without doubt it came from the ends of the bone. The patella was freed. He will have to go back now for another period of fomentations and oil silk.

2. Operation for stone. Bohemian; short and fleshy:

over 70 years of age. He has had trouble in making water for a year; great discomfort for the last five months; pain at the completion of the act of making water, which continues for a little time or until the bladder gradually fills. He urinates every hour and passes a little blood, more when up and about than when sitting or lying.

Dr. B. exhibited a Van Buren's sound and showed also the difficulty of always getting positive evidence even where stone exists. He remarked that the operation had been undertaken without finding stone and even without reaching the bladder; he hoped we should be more fortunate. Etherization in this case was very complete, fifteen minutes having been the time required to put him asleep. The operation was begun and not completed till twenty-five minutes more had elapsed; it illustrated the patience and perseverance needed to succeed sometimes where failure is wholly unlooked for. The difficulty was partly owing to an enlarged prostate and unusual depth of perineum. By a pertinacity worthy of success the stone was finally picked off the floor of the bladder with the forceps over the spot where it lay; by an effort at tossing with the tip of the finger in the rectum, the object was seized and brought forth. The stone was a thick disk with symmetrical rounded edges; weight about four drachms.

March 25. Dr. B. remarked of the old man operated on for stone at the last clinic that he was doing well. Urine passed freely by the natural route. At first the circulation was 96, yesterday it was 80, to-day it is less. The only trouble had been the large distension of the bowels with gas; its passage was promoted by injections of salt and water.

1. Necrosis of bones of the index finger. An incised wound of the first phalangeal joint having failed to unite. Amputation.

2. Necrosis of tibia. Considerable dead bone has been removed in former operations; it has nearly all been cast off; so much new bone has formed that this leg is considerably the larger. The patient having been etherized, elastic bands were applied and the border of the ulcer cut away so as to get at the caries; a sliver of necrosed bone was found and removed; dressed by filling cavity with white wax to keep it open and allow of free discharge.

Dr. B. remarked of the case of caries of the tibia following gunshot wounds presented on the 18th, that the hardness of the bone had heretofore prevented granulations. The medullary cavity was gouged out and the cavity formed by the operation had been frequently washed out and was now granulating very nicely. But to what extent it might fill was uncertain.

3. Injury of the elbow joint. A large boy brought to the hospital yesterday. Considerable swelling. Ether was given. The olecranon was seen to be one side of the middle. The condyles changed their position a little when pressed toward each other. There was a fracture involving the articular surface of the humerus; it extended from the head of the radius upward and inward. Treatment to-day by adhesive straps and pulley, an angular splint not being in readiness.

March 28. 1. Club-foot operation. Babe. Deformity double; the left foot operated on some time ago. To-day the division of the affected tendons of the right. No anæsthetic. The little one's cries during the moment required to complete the operation, eloquently appealed in behalf of chloroform. The wound of the operation will be left to heal, then adhesive plaster to straighten.

2. Abscess of the knee—amputation. An emaciated cachectic youth; inflammation began one month ago about the left knee; abscess burrowing in both directions to the ankle and groin followed. The burrowing has ceased, but he is not improving; it is proposed now to examine, and if necessary, condemn the leg. The pus appears fair, but is very offensive—evidently coming from bone. The head of the tibia is drawn backward and outward. It was found that there was destruction of the synovial membrane and common destruction of cartilage to a greater or less extent. It would never get well. Every preparation having been made, the leg was elevated, and while in this position, the rubber band was wrapped around the thigh ; the elastic roller was not applied on account of the condition of the knee. Amputation was performed at the lower third. Dr. B. remarked that he should tie all slightly bleeding vessels, for tissues infiltrated like these do not allow of contraction like healthy tissue.

3. Varicose veins—compression. The swellings were on the right leg about the knee ; several pins were passed beneath the veins involved and wrapped as in hare-lip sutures ; he is to go to bed and be let alone ; if inflammation should occur, hot water.

4. Anchylosis of the knee. (April 14 and 21.) There was a good deal of soreness after the last operation but not after the first. The limb is affected now by muscular twitching and starting in his sleep. It is proposed to-day to produce extensive division of tendons behind, and to make another effort at straightening ; no more pain or discomfort is apprehended than we have already had. On the completion of the first part of the operation the punctures returned venous blood quite freely. In the last part of the operation, Dr. B. applied his full strength ; the angle of flexion was but very slightly increased. The trial was continued until further effort seemed futile. It is still questionable if he will have any real improvement of the leg—still the attempt has been made.

